# A PD‐L1 Nanobody‐Engineered Synthetic RNA Virus Enhances Hepatocellular Carcinoma Immunotherapy by Interfering PCSK9 and Upregulating MHC‐I

**DOI:** 10.1002/exp2.70226

**Published:** 2026-10-06

**Authors:** Chuyu Huang, Yun Ye, Yi Guo, Guozheng Liao, Linyi Luo, Lingwen Ding, Yingling Miao, Linping Zhao, Yongchang Ouyang, Shiying Li

**Affiliations:** ^1^ Guangdong Provincial Key Laboratory of Molecular Target & Clinical Pharmacology, the NMPA and State Key Laboratory of Respiratory Disease, the School of Pharmaceutical Sciences Guangzhou Medical University Guangzhou China; ^2^ GMU‐GIBH Joint School of Life Sciences Guangzhou Medical University Guangzhou China; ^3^ Department of Pathology, Yong Loo Lin School of Medicine National University of Singapore Singapore Singapore

**Keywords:** gene therapy, immunotherapy, MS2 virus‐like particles

## Abstract

Hepatocellular carcinoma (HCC) is characterized by low expression of major histocompatibility complex class I (MHC‐I), which significantly limits the efficacy of immunotherapy. In this work, bioinformatic analysis finds that elevated PCSK9 in HCC correlates with reduced T cell activity and poor prognosis. Based on these discoveries, a PD‐L1 nanobody‐engineered synthetic RNA virus (αPDL1‐VLPs/shPCSK9) is fabricated to inhibit PCSK9, thereby restoring MHC‐I expression to enhance HCC immunotherapy. The αPDL1‐VLPs/shPCSK9 is produced using MS2 virus‐like particles (VLPs) in *Escherichia coli* (*E. coli*), displaying PD‐L1 nanobodies and encapsulating PCSK9‐downregulating shRNA. Notably, αPDL1‐VLPs/shPCSK9 upregulates MHC‐I to enhance cytotoxic T lymphocytes (CTLs) recognition of tumor cells while promoting M2‐to‐M1 macrophages polarization, thereby boosting immunotherapy. In vivo studies indicate that αPDL1‐VLPs/shPCSK9 exhibits HCC tumor‐targeting behavior, suppressing primary tumor growth while avoiding systemic toxicity. Immunological analysis confirms that αPDL1‐VLPs/shPCSK9 enhances CTLs activation and infiltration, triggering systemic antitumor immunity to eradicate distant tumors. Overall, these findings highlight αPDL1‐VLPs/shPCSK9 as a promising therapeutic strategy for reversing the multiple immunosuppressive characteristics of HCC, providing a streamlined yet comprehensive approach to potentiate immunotherapy.

## Graphical abstract

A PD‐L1 nanobody‐engineered synthetic RNA virus (αPDL1‐VLPs/shPCSK9) is fabricated to inhibit PCSK9 and upregulate MHC‐I to facilitate T cells recognition of hepatocellular carcinoma (HCC). Moreover, αPDL1‐VLPs/shPCSK9 can recognize PD‐L1 and block the PD‐1/PD‐L1 pathway to prevent immune evasion, while promoting M2‐to‐M1 macrophages polarization, leading to a synergistic activation of systemic antitumor responses for the elimination of primary and distant tumors.

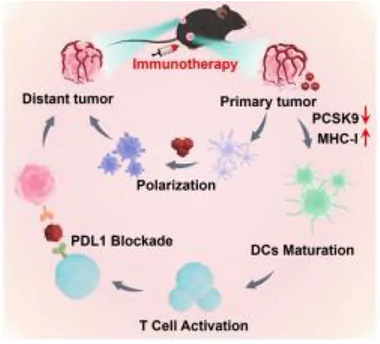

## 1 Introduction

Hepatocellular carcinoma (HCC) is a highly prevalent malignancy, with a dismal 5‐year survival rate of 18% across all stages. Current surgical and locoregional therapies provide limited improvement in patient outcomes, and the immunosuppressive nature of HCC further complicates effective treatment [1]. HCC evades immune surveillance by exploiting multiple immunosuppressive mechanisms that not only promote tumor progression but also hinder the efficacy of systemic immunotherapies [2, 3]. Among these mechanisms, the overexpression of programmed death ligand 1 (PD‐L1) on tumor cells allows interaction with programmed death 1 (PD‐1) on T cells, leading to T cell exhaustion and impaired proliferation [4, 5]. Although immune checkpoint inhibitors targeting PD‐L1 have shown promise in clinical settings, the objective response rates in advanced HCC patients remain modest, ranging from 15% to 20% [6, 7]. Among which, the major barrier to effective antitumor immunity is the downregulation or complete loss of major histocompatibility complex class I (MHC‐I) expression, with 40%–90% of tumors exhibiting MHC‐I deficiencies, impairing antigen presentation to cytotoxic T lymphocytes (CTLs) [8]. This issue is further exacerbated by chronic hepatitis B (HBV) and hepatitis C (HCV) infections, which contribute to MHC‐I degradation through genome integration or signal peptide peptidase activity, further suppressing immune recognition [9, 10]. Therefore, restoring MHC‐I expression has emerged as a critical strategy to enhance immune responses in HCC therapy [11].

Recent studies have identified that cholesterol modulation enhances CTLs’ proliferation and effector function [12]. In this context, proprotein convertase subtilisin/kexin type 9 (PCSK9), a protease responsible for cholesterol homeostasis and MHC‐I expression, is overproduced in HCC, offering a potential target to potentiate immune checkpoint blockade therapy [13, 14, 15]. Most marketed PCSK9 inhibitors are monoclonal antibodies, which face challenges such as high manufacturing costs and complex production processes. RNA‐based therapies present a promising approach for targeting PCSK9 due to their ability to modulate gene expression with high specificity [16, 17]. However, challenges such as poor cellular uptake due to RNA's negative charge, vulnerability to degradation by RNases, and lack of cell‐specific delivery limit their therapeutic efficacy and increase the risk of off‐target effects, especially in genes like PCSK9 that are involved in normal physiological functions [18]. To overcome these obstacles, a suitable RNA delivery system is essential for efficient PCSK9 interference in immune regulation and HCC therapy [19].

Notably, an ideal RNA delivery system must simultaneously achieve high transfection efficiency, excellent biosafety, scalable manufacturing, and robust storage stability. Taking the most clinically advanced delivery platform, lipid nanoparticles (LNPs), as an example, despite their successful application in COVID‐19 mRNA vaccines, they still face key limitations, including high production cost, challenges in large‐scale manufacturing, suboptimal storage and transportation stability, and the need for complex surface engineering to achieve targeted delivery [20, 21]. Viral‐like particles (VLPs), nanoscale assemblies of viral proteins lacking genetic material, offer a compelling alternative due to their uniformity, biocompatibility, and reduced toxicity [22, 23]. VLPs can be genetically modified or chemically conjugated with targeting ligands to facilitate cell‐specific drug delivery, while their internal cavities protect therapeutic nucleic acids from degradation [24]. However, traditional chemical modification and conjugation methods are inefficient, complex, and costly, limiting their applications. Therefore, the one‐step synthesis of functional VLPs is particularly critical. One notable example is the bacteriophage MS2 VLP, which consists of 180 coat proteins (CP) capable of self‐assembling into icosahedral structure [25, 26, 27]. These CP display peptides or nanobodies on the exterior surface, thereby achieving targeted functions. Additionally, these CP exhibit high adaptability. Under the effect of stem‐loop structure, they promote their own arrangement and assembly, eventually forming a polyhedral VLP with a cavity structure. During the assembly process, drugs, genes, and other substances, after binding with the stem‐loop, can be effectively encapsulated inside the VLP [28, 29]. More importantly, VLPs do not contain the bacteriophage MS2 genome, which allows the CP to retain its ability to activate the immune system as an adjuvant, while also reducing potential safety risks. The well‐defined structure, efficient RNA packaging, and high transfection capability of MS2 VLPs endow them with virus‐like properties, making them highly suitable for RNA‐based therapeutic applications [30].

In this study, bioinformatic analyses verified that elevated PCSK9 levels correlated with diminished T cell activity and poor prognoses in HCC patients. In light of the challenges posed by HCC, particularly the role of PCSK9 in immune evasion, a PD‐L1 nanobody‐engineered synthetic RNA virus (designated as αPDL1‐VLPs/shPCSK9) was fabricated to enhance HCC immunotherapy through the interfering of PCSK9 (Scheme 1A). To construct the αPDL1‐VLPs/shPCSK9, MS2 VLPs were synthesized in *Escherichia coli* (*E. coli*), exhibiting high affinity for PD‐L1, thus facilitating targeted delivery to HCC cells and enabling immune checkpoint blockade. Through optimization, including screening for the appropriate sequence localization of shPCSK9 and stem‐loop structures, we achieved a robust virus‐like nanoplatform characterized by PD‐L1 specificity and efficient RNA packaging. Notably, αPDL1‐VLPs/shPCSK9 effectively downregulated PCSK9 expression, simultaneously enhancing MHC‐I levels, which were crucial for T cell recognition of tumor cells. Furthermore, our findings demonstrated that MS2 VLPs induced the polarization of M2 macrophages to the pro‐inflammatory M1 phenotype, thereby synergistically enhancing immune activation. In vivo studies corroborated the excellent tumor‐targeting capabilities of αPDL1‐VLPs/shPCSK9, which suppressed primary tumor growth without eliciting systemic side effects. Additionally, immunological assessments indicated that this platform promoted CTLs activation and infiltration, facilitating the eradication of distant tumors through the engagement of systemic antitumor immunity (Scheme 1B). Collectively, this study presented a straightforward yet multifaceted approach to co‐delivering nanobodies and RNA, providing a sophisticated mechanism to harness therapeutic feedback and augment HCC immunotherapy.

**SCHEME 1:**
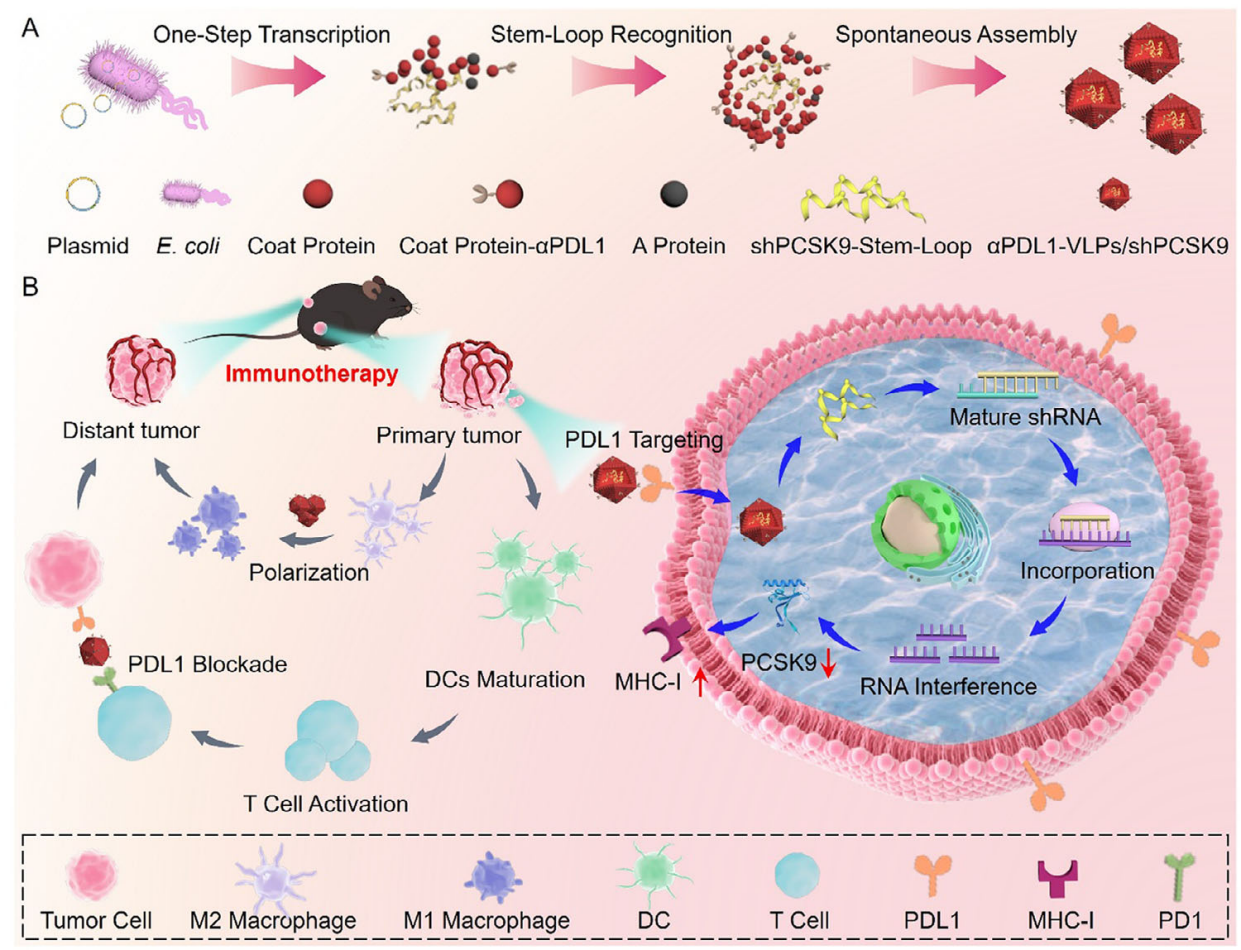
Schematic illustration of the preparation of αPDL1‐VLPs/shPCSK9 and the proposed mechanism to enhance HCC immunotherapy. (A) The PD‐L1 nanobody‐engineered synthetic RNA virus (αPDL1‐VLPs/shPCSK9) was synthesized in a one‐step process through *E. coli* plasmid transfection, encoding coat protein, αPDL1‐fused coat protein, A protein, and shPCSK9‐stem‐loop. (B) αPDL1‐VLPs/shPCSK9 actively accumulated in tumors through PD‐L1 recognition, while also blocking the PD‐1/PD‐L1 pathway to prevent immune evasion. Furthermore, αPDL1‐VLPs/shPCSK9 downregulated PCSK9 and upregulated MHC‐I to facilitate T cells recognition of tumors. Moreover, αPDL1‐VLPs/shPCSK9 promoted the polarization of M2 macrophages toward the pro‐inflammatory M1 phenotype, resulting in synergistic activation of systemic antitumor immunity and enhanced clearance of distant tumors.

## 2 Results and Discussion

### 2.1 Preparation and Characterization of αPD‐L1 Nanobody and αPDL1‐VLPs

While PD‐L1‐targeting antibodies have demonstrated efficacy in cancer therapy, their clinical application is often limited by high production costs, suboptimal stability, and poor tissue penetration [31, 32]. Compared to full‐length antibodies, nanobodies exhibit several remarkable characteristics, including deeper tissue penetration, high structural stability, and facile engineering and production processes [33, 34, 35]. As depicted in Figure S1A, a plasmid encoding the PD‐L1‐specific nanobody (αPD‐L1) was constructed. ELISA results showed that αPD‐L1 bound specifically to PD‐L1, with an EC50 of 0.2 ± 0.06 µg mL^−1^ (Figure 1A,B). A concentration‐dependent increase in absorbance further confirmed the specific binding of αPD‐L1 to PD‐L1 (Figure 1C). These findings indicated that plasmid‐produced αPD‐L1 possessed high affinity and specificity for PD‐L1. Even so, the rapid pharmacokinetics of nanobodies might limit their therapeutic efficacy. To address this challenge, bacteriophage MS2 VLPs were designed to display PD‐L1 nanobodies, enabling efficient nanobody delivery. Among which, the CP and a small number of A proteins (AP) of bacteriophage MS2 could self‐assemble into VLPs guided by stem‐loop structures, mimicking a synthetic process that emulated viral assembly. These proteins were arranged in an orderly fashion to form an icosahedron with a hollow structure. Then, through genetic engineering, the αPD‐L1 sequence was inserted into the MS2 VLP‐expressing plasmid, resulting in αPD‐L1‐displaying VLPs (αPDL1‐VLPs) after colony selection, large‐scale culture, and purification (Figures S1B and S2). Transmission electron microscopy (TEM) and dynamic light scattering analyses revealed that αPDL1‐VLPs were approximately spherical with a hydrodynamic diameter of 47.36 ± 6.40 nm, facilitating tumor accumulation via the enhanced permeability and retention (EPR) effect (Figure 1D,E). In addition, the particle size remained stable over five days, indicating good stability (Figure 1F). Notably, the αPD‐L1 displayed on the VLPs surface retained its specific binding ability to PD‐L1, with 41‐fold higher specificity compared to BSA (Figure 1G). The EC50 of αPD‐L1‐VLPs was 1.8 ± 0.5 µg mL^−1^, slightly higher than that of free αPD‐L1, but the concentration‐dependent binding characteristic remained unaffected (Figure 1H,I). Quantification of αPD‐L1 (32 kDa) and CP (13 kDa) demonstrated that each αPDL1‐VLP contained approximately 109 ± 9 copies of αPD‐L1 (Figure S3). Taken together, these results indicated that displaying nanobodies on VLP surfaces was a feasible and efficient strategy for nanobody delivery while maintaining their specificity.

**FIGURE 1:**
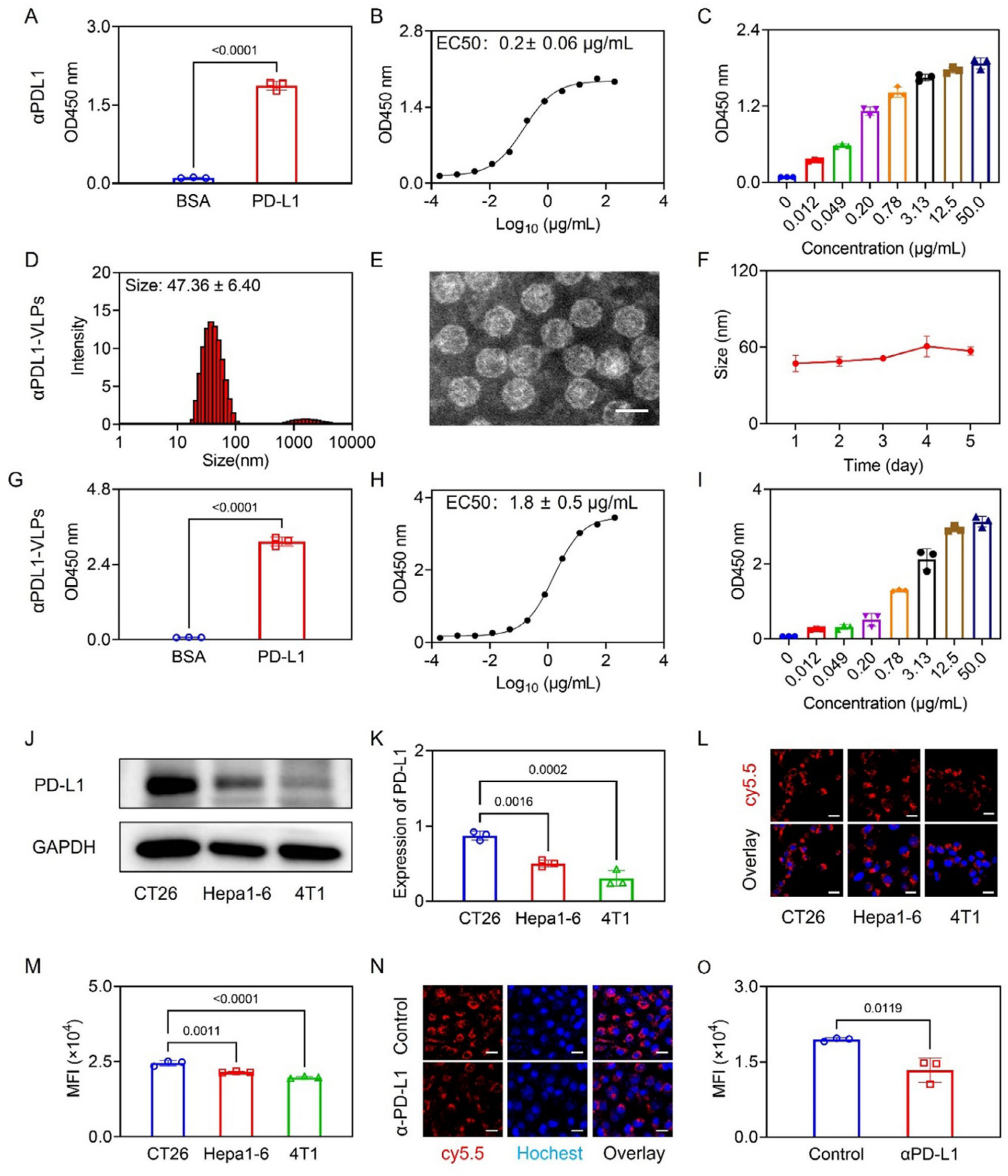
Fabrication and characterization of αPDL1‐VLPs. (A) Assessment of the specific PD‐L1 targeting capability of αPD‐L1 in comparison to BSA (n = 3). (B) Determination of EC50 for the binding affinity of αPD‐L1 to PD‐L1. (C) Evaluation of PD‐L1 targeting efficacy across various concentrations of αPD‐L1 (n = 3). (D) Hydrodynamic diameter, (E) TEM image and (F) particle stability of αPDL1‐VLPs. Scale bar: 25 nm. (G) The PD‐L1 specific targeting capability of αPDL1‐VLPs (n = 3). (H) EC50 and (I) PD‐L1 targeting capability at varying concentrations of αPDL1‐VLPs (n = 3). (J) Western blot analysis of PD‐L1 expression in CT26, Hepa1‐6, and 4T1 cells. (K) Quantification of PD‐L1 expression levels in different cell lines (n = 3). (L) CLSM images of CT26, Hepa1‐6, and 4T1 cells treated with αPDL1‐VLPs@cy5.5 for 6 h. Scale bar: 20 µm. (M) Quantification of mean fluorescence intensity (MFI) of αPDL1‐VLPs@cy5.5 in different cell lines at 6 h (n = 3). (N) CLSM images and (O) quantification of MFI in Hepa1‐6 cells pre‐treated with or without PD‐L1 antibody to block PD‐L1 binding sites, followed by treatment with αPDL1‐VLPs@cy5.5 (n = 3). Scale bar: 20 µm. Statistical analyses between the indicated groups were performed using Student's t test (A, G and O) and one‐way ANOVA analysis (K and M). *p < 0.05, **p < 0.01, ***p < 0.001, ****p < 0.0001.

### 2.2 PD‐L1 Targeted Delivery of αPDL1‐VLPs

To elucidate the targeting capability of αPDL1‐VLPs toward PD‐L1 on liver cancer cells, comparative analyses were conducted using CT26 cells with high PD‐L1 expression and 4T1 cells with low PD‐L1 expression as controls (Figure 1J). Among which, PD‐L1 expression in CT26 cells was 1.74 times higher than in Hepa1‐6 cells, whereas 4T1 cells exhibited PD‐L1 levels at 0.61 times that of Hepa1‐6 cells (Figure 1K). Subsequently, the cellular internalization efficiency of cy5.5‐labeled αPDL1‐VLPs (αPDL1‐VLPs@cy5.5) was assessed across these cell lines. As shown in Figure 1L,M, fluorescence intensity in CT26 cells was significantly greater than that in Hepa1‐6 and 4T1 cells, indicating efficient binding and uptake of αPDL1‐VLPs by cells with more PD‐L1 expressions. Furthermore, pretreatment with a PD‐L1 antibody diminished the targeting efficacy of αPDL1‐VLPs, as evidenced by reduced fluorescence intensity in the pretreated cells (Figure 1N,O). These experiments in cells with varying PD‐L1 expression levels not only indicated the therapeutic relevance of αPDL1‐VLPs but also validated the potential in treating PD‐L1 expressing HCC.

### 2.3 Bioinformatics Analysis of PCSK9 and Its Role in HCC

HCC is a life‐threatening cancer that often exhibits limited responsiveness to anti‐PD‐L1 therapies. Identifying immune‐potentiating targets and developing synergistic treatment strategies could enhance therapeutic outcomes for HCC patients. To identify immunosuppressive factors that might diminish the efficacy of αPD‐L1 therapy in HCC patients, bioinformatic analyses had been conducted to screen for synergistic therapeutic targets. As shown in Figure 2A, PCSK9 expression was significantly elevated in HCC tissues compared to normal tissues (*p* < 0.001). PCSK9 was known to promote the degradation of low‐density lipoprotein receptors (LDLR), reducing the uptake of liver cholesterol [33, 34, 35]. This reduction could lead to elevated blood cholesterol levels, potentially impairing liver function and creating a favorable environment for tumor development [37]. Notably, high PCSK9 expression had been associated with altered immune cell infiltration, showing a strong correlation with the activation states of CD4^+^ and CD8^+^ T cells (Figure S4A). These findings suggested that PCSK9 might compromise liver cancer treatment efficacy by reshaping immune status within the tumor microenvironment. Survival analysis further revealed a strong correlation between high PCSK9 expression and poor prognosis in liver cancer patients (Figure S4B). Over a 120‐month follow‐up period, patients with elevated PCSK9 expression exhibited significantly lower overall survival rates compared to those with low expression (*p* = 0.019). This finding highlighted PCSK9's potential as a prognostic marker and suggested that it might contribute to the malignant progression of HCC through multiple mechanisms. Consequently, PCSK9 had emerged not only as a potential biomarker for liver cancer but also as a promising target for enhancing immunotherapies.

**FIGURE 2:**
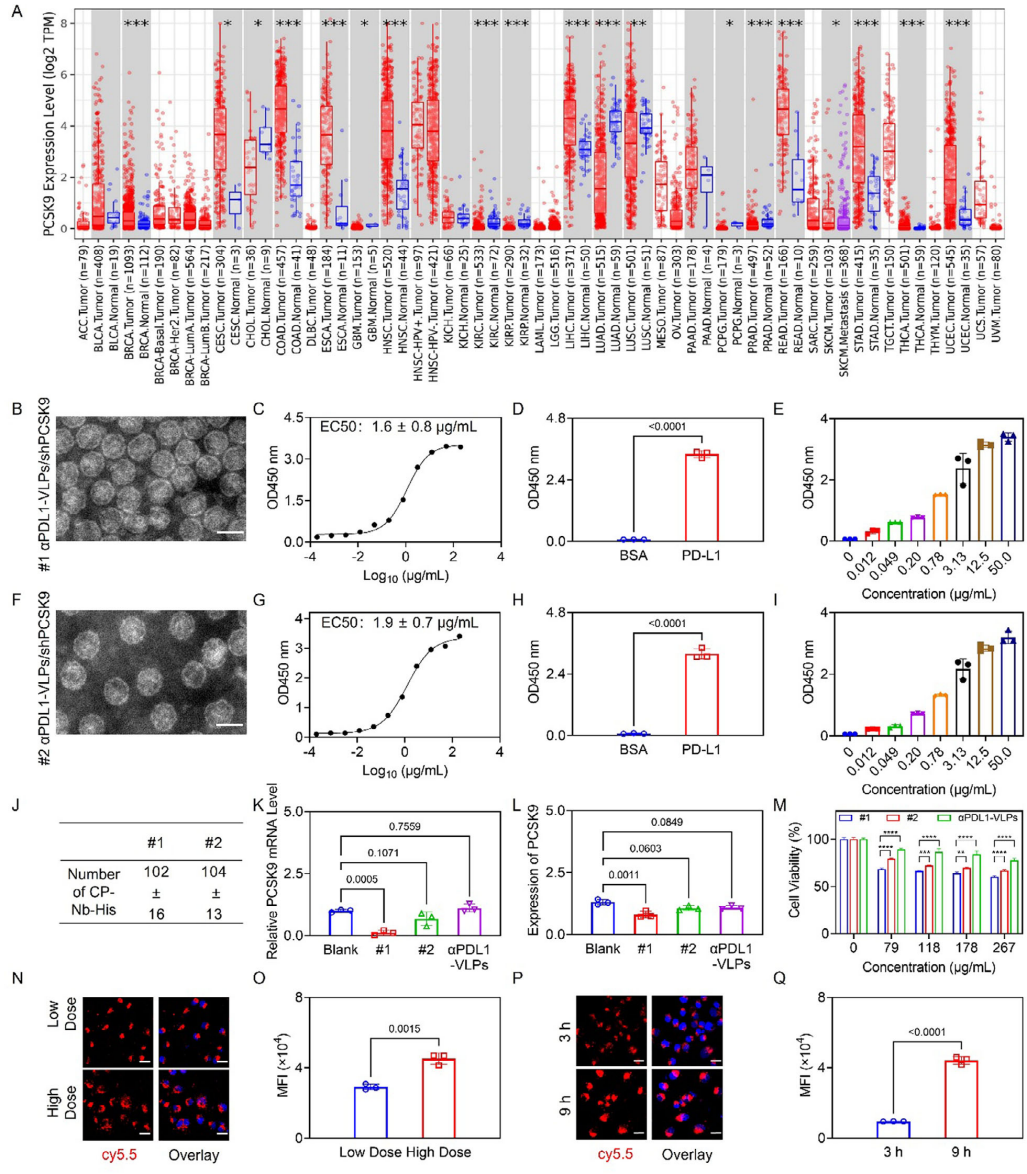
Bioinformatic analysis of HCC and the fabrication of αPDL1‐VLPs/shPCSK9. (A) Comparison of PCSK9 expression levels across various human tumors, with tumor samples (red) and normal tissue samples (blue). (B) The TEM image of #1 αPDL1‐VLPs/shPCSK9. Scale bar: 25 nm. (C) Determination of the EC50 for binding of #1 αPDL1‐VLPs/shPCSK9 to PD‐L1. (D) Evaluation of the PD‐L1 targeting capability of #1 αPDL1‐VLPs/shPCSK9 in comparison to BSA (n = 3). (E) PD‐L1 targeting capability at different concentrations of #1 αPDL1‐VLPs/shPCSK9 (n = 3). (F) The TEM image, (G) EC50 of PD‐L1 binding, (H) PD‐L1 specific targeting capability and (I) PD‐L1 targeting capability at different concentration of #2 αPDL1‐VLPs/shPCSK9 (n = 3). Scale bar: 25 nm. (J) The number of CP containing αPD‐L1 in #1 αPDL1‐VLPs/shPCSK9 and #2 αPDL1‐VLPs/shPCSK9. (K) Real‐time PCR analysis of PCSK9 mRNA level and (L) quantitative analysis of PCSK9 protein expression in Hepa1‐6 cells after treatment with #1 αPDL1‐VLPs/shPCSK9, #2 αPDL1‐VLPs/shPCSK9 and αPDL1‐VLPs (n = 3). (M) CCK8 assay evaluating the viability of Hepa1‐6 cells treated with #1 αPDL1‐VLPs/shPCSK9, #2 αPDL1‐VLPs/shPCSK9, and αPDL1‐VLPs. (N) CLSM images and (O) quantitative fluorescence analysis of Hepa1‐6 cells following treatment with different concentrations of #1 αPDL1‐VLPs/shPCSK9 for 9 h (n = 3). Scale bar: 20 µm. (P) CLSM images and (Q) quantitative fluorescence analysis of Hepa1‐6 cells following treatment with #1 αPDL1‐VLPs/shPCSK9 for 3 or 9 h (n = 3). Scale bar: 20 µm. Statistical analyses between the indicated groups using Wilcoxon test (A), Student's t test (D, H, O, Q) and one‐way ANOVA analysis (K, L and M). *p < 0.05, **p < 0.01, ****p < 0.0001.

### 2.4 Preparation and Characterization of αPDL1‐VLPs/shPCSK9

RNA interference (RNAi) is a powerful method for gene silencing. However, its practical application is hindered by the inherent instability of RNA molecules and their susceptibility to nuclease degradation. Moreover, the hydrophobic lipid bilayer of cell membranes acts as a barrier to charged molecules, impeding the effective penetration of negatively charged RNA into cells [38]. Inspired by the success of αPDL1‐VLPs, we aimed to develop a one‐step synthesis strategy to fabricate PD‐L1 nanobody‐engineered and PCSK9 shRNA‐encapsulated VLPs (αPDL1‐VLPs/shPCSK9). This approach harnessed synthetic RNA virus packaging mechanisms, optimizing RNA payload delivery efficiency and amplifying PCSK9‐silencing effects. Considering potential effects on expression efficiency, the optimal insertion site for the shPCSK9 gene sequence in the plasmid was identified [39]. Therefore, two plasmids were designed to incorporate shPCSK9 into αPDL1‐VLPs. In one plasmid, the stem‐loop sequence was positioned downstream of shPCSK9 and designated as #1 αPDL1‐VLPs/shPCSK9, while in the other, the stem‐loop was placed upstream and designated as #2 αPDL1‐VLPs/shPCSK9 (Figure S5). Due to the connection between shPCSK9 and the stem‐loop, shPCSK9 was encapsulated within the VLPs along with the stem‐loop during CP assembly. Subsequently, these plasmids were transfected into competent *E. coli*, and single clones were selected on solid media, expanded, isolated, and purified (Figure S6). Electrophoresis and protein staining samples from various culture stages revealed that His‐nickel affinity chromatography isolated CP at 13 kDa, a His‐tagged CP fused with αPD‐L1 at 32 kDa and AP at 44 kDa (Figure S7). This indicated that the plasmid had been successfully expressed. As shown in Figures S8A and 2B, TEM images revealed that #1 αPDL1‐VLPs/shPCSK9 exhibited a polyhedral morphology resembling a sphere, with a hydrodynamic diameter of 50.16 ± 3.21 nm. Moreover, the incorporation of shPCSK9 did not compromise the PD‐L1 targeting capability of surface αPD‐L1, as the EC50 of #1 αPDL1‐VLPs/shPCSK9 (1.6 ± 0.8 µg mL^−1^) was comparable to that of αPDL1‐VLPs (Figure 2C–E). When the stem‐loop was positioned upstream of shPCSK9, the particle size of #2 αPDL1‐VLPs/shPCSK9 was 50.24 ± 1.82 nm, similar to #1 αPDL1‐VLPs/shPCSK9 (Figure S8B). Additionally, #2 αPDL1‐VLPs/shPCSK9 exhibited comparable morphology and PD‐L1 targeting efficacy, with an EC50 of 1.9 ± 0.7 µg mL^−1^ (Figure 2F–I). These results demonstrated that the size of VLPs was not closely related to the type or quantity of the genes contained within, and the presence and position of shPCSK9 gene sequence did not affect the stem loop's ability to induce CP assembly. Next, western blot analysis using a His‐tag antibody demonstrated that not all CP molecules carried the His‐tag and αPD‐L1, and untagged CP at 13 kDa was undetectable (Figure S9). This suggested that the plasmid might have undergone stop codon read through in *E. coli*, leading to the display of nanobodies on the surface of certain CP. This partial display phenomenon reduced steric hindrance, thereby decreasing the possibility of VLPs assembly failure. Afterward, quantitative analysis indicated that each #1 αPDL1‐VLPs/shPCSK9 contained 102 ± 16 copies of αPD‐L1, while each #2 αPDL1‐VLPs/shPCSK9 contained 104 ± 13 copies (Figures 2J and S10). Physicochemical characterization, including particle size and protein concentration, was performed for each batch to confirm consistency within acceptable error ranges. Moreover, after treatment with RNase A and DNase I, genes remained detectable in both #1 and #2 αPDL1‐VLPs/shPCSK9 (Figure S11), suggesting that the VLPs protected shRNA from nuclease degradation and facilitated its delivery into cells to exert its function.

After preparation and characterization of αPDL1‐VLPs/shPCSK9, further evaluation was performed to assess their impact on PCSK9 expression. As shown in Figure 2K, the mRNA expression level of PCSK9 following #1 αPDL1‐VLPs/shPCSK9 treatment was 0.11 times that of the control group. In contrast, the mRNA level after #2 αPDL1‐VLPs/shPCSK9 treatment was 0.68 times that of the control group. Furthermore, #1 αPDL1‐VLPs/shPCSK9 remarkably reduced PCSK9 protein expression to 62% of that observed in the non‐treatment group. As expected, #2 αPDL1‐VLPs/shPCSK9 downregulated PCSK9 expression to only 18%, a change that was not statistically significant (Figures 2L and S12). Cytotoxicity assays demonstrated that #1 αPDL1‐VLPs/shPCSK9, which more effectively regulated PCSK9, exhibited enhanced tumor cell‐killing activity (Figure 2M). The above results indicated that positioning the stem‐loop structure in front of the shPCSK9 gene sequence diminished its silencing efficacy. Therefore, subsequent experiments utilized #1 αPDL1‐VLPs/shPCSK9 as the therapeutic agent. In addition, the particle size of #1 αPDL1‐VLPs/shPCSK9 was measured on day 7 and day 14, yielding values of 61.80 ±1.05 nm and 47.76 ±1.01 nm, respectively (Figure S13). Notably, the particle size showed only minor fluctuation and remained largely stable over the observed period. This stability supported both long‐term storage and safety for sustained administration, underscoring one of the key advantages of this system as a drug delivery platform. Meanwhile, αPDL1‐VLPs/shPCSK9 demonstrated targeting specificity for Hepa1‐6 cells, with cellular uptake increasing in a concentration‐ and time‐dependent manner (Figure 2N–Q), a critical feature for efficient gene silencing and sustained therapeutic effects through enhanced RNA payload delivery. Additionally, the antitumor effect of αPDL1‐VLPs/shPCSK9 at the cellular level was evaluated using Sorafenib, a first‐line targeted therapy for HCC, as a positive control. Through targeted silencing of PCSK9 gene expression, αPDL1‐VLPs/shPCSK9 effectively induced early apoptosis of HCC cells, and demonstrated superior tumor‐killing activity compared with sorafenib (Figure S14).

### 2.5 Immunomodulatory Effects of αPDL1‐VLPs/shPCSK9

Before assessing the immunotherapeutic effect of αPDL1‐VLPs/shPCSK9, the immunogenicity of the VLP carrier was first evaluated. It was reported that VLPs could promote the expression of MHC‐I molecules through immune‐stimulating mechanisms [40, 41]. It was intriguing to find that αPDL1‐VLPs increased MHC‐I expression, with this effect being further enhanced following treatment with αPDL1‐VLPs/shPCSK9 (Figure 3A). This was owing to the capsid protein of the VLP possessing intrinsic immunostimulatory activity, which directly enhanced MHC‐I expression on tumor cells. Moreover, shPCSK9 further amplified this effect by silencing PCSK9, a protein known to bind MHC‐I and promote its lysosomal degradation. Additionally, flow cytometry analysis revealed that αPDL1‐VLPs/shPCSK9 treatment increased the percentage of MHC‐I positive cells to 19.7%, compared to 14.1% in the αPDL1‐VLPs treatment group (Figure 3B). Meanwhile, the mean fluorescence intensity of MHC‐I in the αPDL1‐VLPs/shPCSK9 treatment group was 1.3‐fold higher than that in the αPDL1‐VLPs treatment group, confirming that PCSK9 downregulation enhanced MHC‐I expression (Figure 3C). The upregulation of MHC‐I expression facilitated CD8^+^ T cell recognition of tumor cells, thereby enhancing the T cell‐mediated immune response [42]. Furthermore, treatment with αPDL1‐VLPs/shPCSK9 reduced Arginase‐1 (Arg) expression in macrophages to 0.51 times that of untreated cells. Arg competed with the inducible nitric oxide synthase (iNOS) of M1 macrophages for the common substrate L‐arginine. By converting L‐arginine into ornithine and urea, Arg decreased iNOS‐derived nitric oxide production and suppressed pro‐inflammatory amplification. Arg and iNOS were typically used as markers for macrophage classification. Concurrently, αPDL1‐VLPs/shPCSK9 significantly upregulated iNOS expression in M1 macrophages, reaching levels 31 times higher than those in M2 macrophages (Figure 3D–F). To further confirm its polarization effect, the proportions of M2 (CD206) and M1 (CD80) macrophages were analyzed using flow cytometry. Compared to untreated controls, the treatment reduced M2 macrophages prevalence from 45.2% to 33.7%, accompanied by a 56.0% decrease in MFI of the M2 specific marker (Figure 3G,H). Conversely, αPDL1‐VLPs/shPCSK9 treatment increased the proportion of M1 macrophages from 4.54% to 14.2% compared with non‐treatment group, of which the corresponding marker MFI elevated 4.1‐fold relative to the non‐treatment group (Figure 3I–J). All of these findings emphasized that αPDL1‐VLPs/shPCSK9 possessed additional immunomodulatory effects, facilitating M2‐to‐M1 macrophages polarization and reversing the immunosuppressive tumor microenvironment.

**FIGURE 3:**
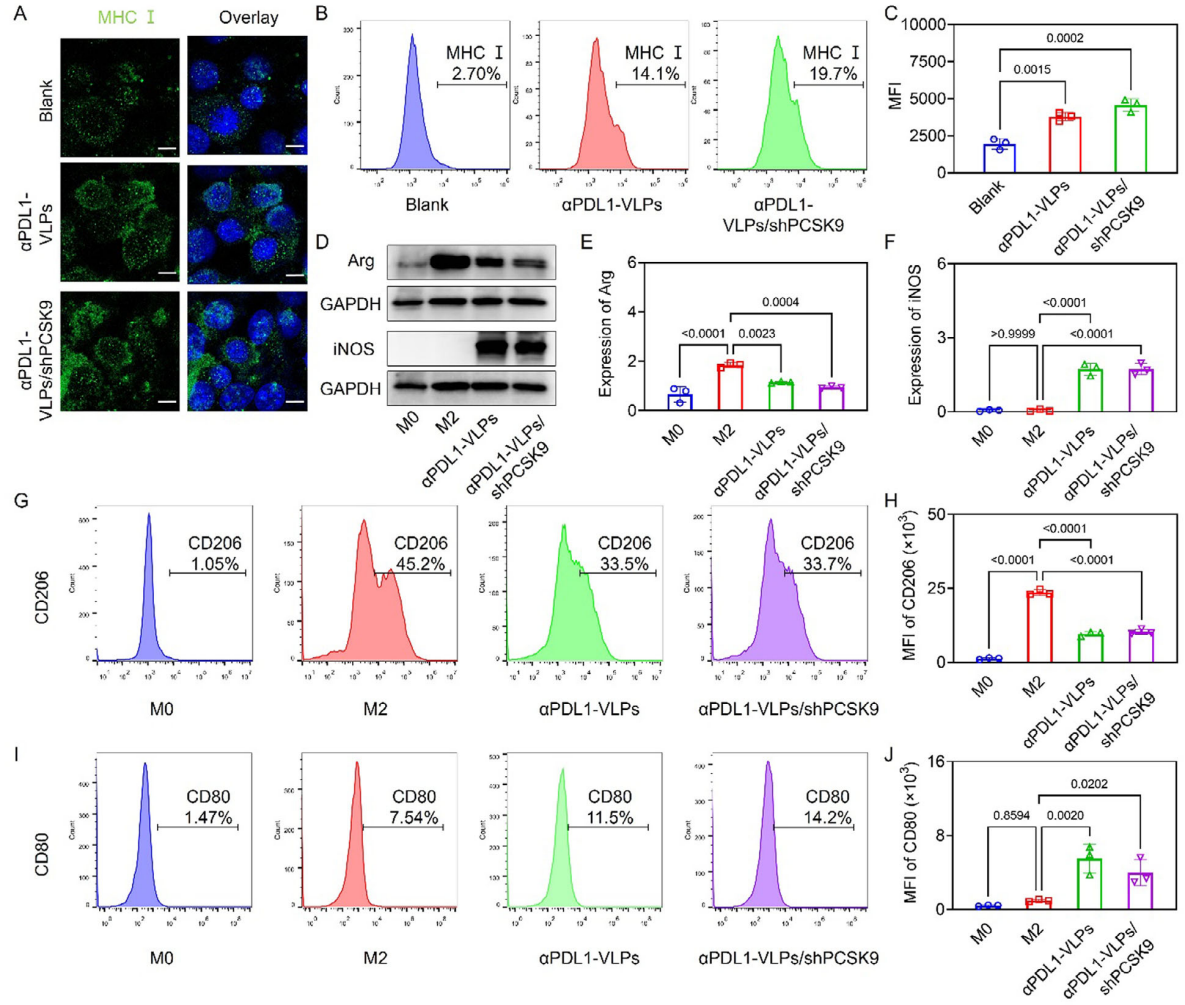
Immunomodulatory effects of αPD‐L1‐VLPs/shPCSK9. (A) Immunofluorescence staining analysis and (B) flow cytometry assessment of MHC‐I after treatment with αPDL1‐VLPs and αPDL1‐VLPs/shPCSK9. Scale bar: 10 µm. (C) Quantitative fluorescence analysis of MHC‐I after various treatments (n = 3). (D) Western blot analysis exhibiting the expression levels of Arg and iNOS in M2 macrophages after treatment with αPD‐L1‐VLPs and αPD‐L1‐VLPs/shPCSK9. Quantification of (E) M2 macrophage marker (Arg) and (F) M1 macrophage marker (iNOS) expression following different treatments (n = 3). (G) Flow cytometry analysis and (H) MFI of M2 macrophage marker (CD206) expression following treatment with αPDL1‐VLPs and αPDL1‐VLPs/shPCSK9 (n = 3). Analysis of M1 macrophage marker (CD80) expression via (I) flow cytometry and (J) MFI after different treatments (n = 3). Statistical analysis between the indicated groups was performed using one‐way ANOVA analysis. *p < 0.05, **p < 0.01, ***p < 0.001, ****p < 0.0001.

### 2.6 Tumor Targeting Suppression of αPDL1‐VLPs/shPCSK9 In Vivo

The efficient targeting ability, gene regulation function, and immune modulation of αPDL1‐VLPs/shPCSK9 motivated us to further evaluate its antitumor effects in vivo. To assess its biodistribution, αPDL1‐VLPs/shPCSK9 was labeled with cy5.5 and administered to Hepa1‐6 tumor‐bearing mice. As shown in Figure 4A and S15, αPDL1‐VLPs/shPCSK9@cy5.5 exhibited obvious tumor accumulation and prolonged retention. After 24 h, mice were euthanized, and their heart, liver, spleen, lung, kidney, and tumor tissues were collected for *ex vivo* fluorescence imaging. Results showed distinct fluorescent signals in tumor tissues, confirming the tumor‐targeting capability of αPDL1‐VLPs/shPCSK9. Quantitative analysis of fluorescence intensity further revealed that, apart from the high accumulation in tumors, the remaining agents were predominantly distributed in the liver (Figure S16), indicating a primarily hepatic metabolic pathway. This efficient tumor‐specific enrichment and clear hepatic clearance route provided a promising basis for the precision treatment of liver cancer.

**FIGURE 4:**
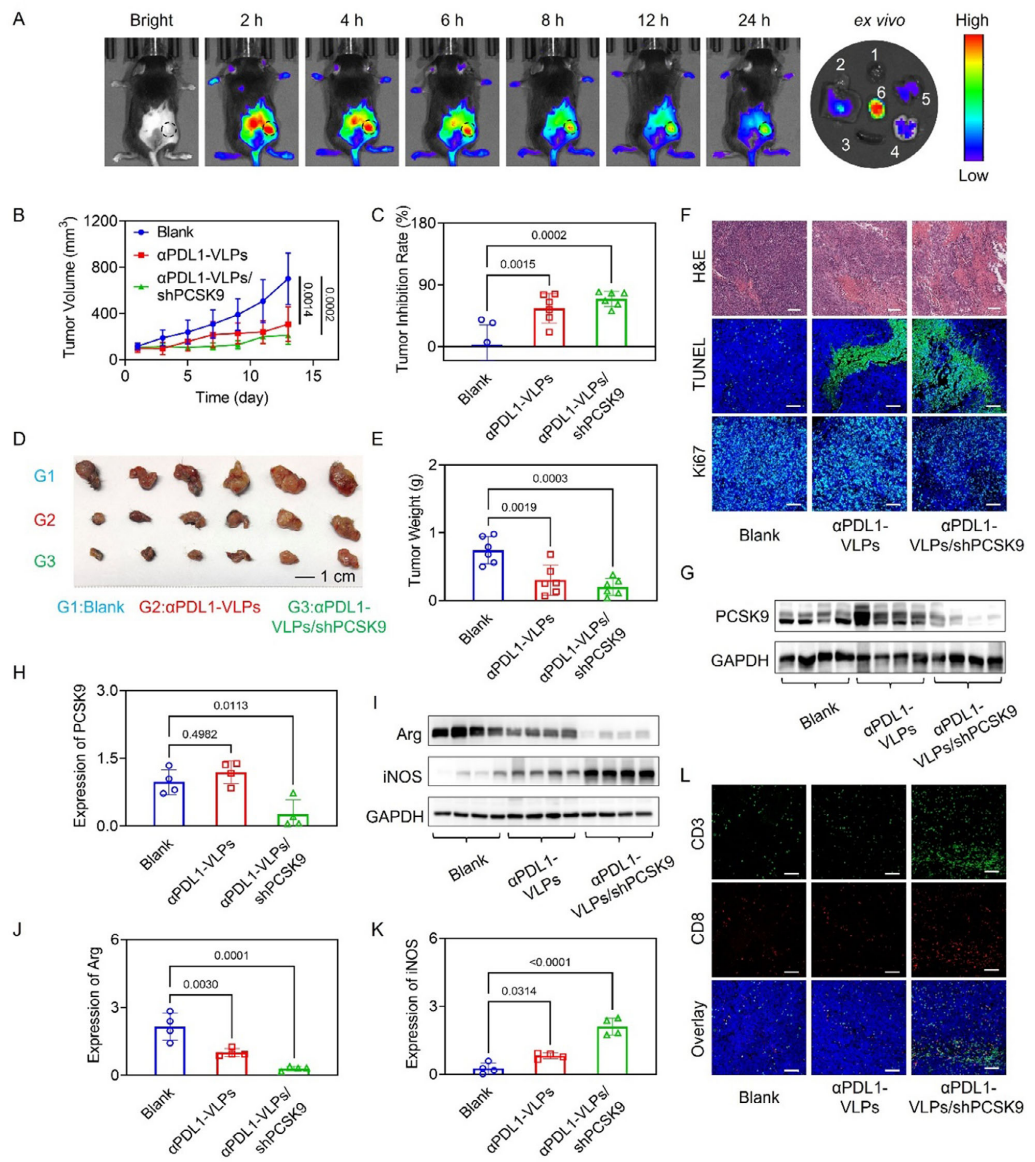
Antitumor effects of αPDL1‐VLPs/shPCSK9 in vivo. (A) Biodistribution analysis of αPDL1‐VLPs/shPCSK9@cy5.5 using real time fluorescence imaging after intravenous injection. 1–6 respectively represented the ex vivo images of heart, liver, spleen, lung, kidney and tumor tissues. (B) Tumor volume curves of mice after treatment by αPDL1‐VLPs, αPDL1‐VLPs/shPCSK9 over a period of 13 days (n = 6). (C) Tumor inhibition rate, (D) harvested tumor tissues and (E) mean tumor weight after various treatments (n = 6). (F) H&E staining, TUNEL and Ki67 immunofluorescence images of isolated tumor tissues. Scale bar: 100 µm. (G) PCSK9 expression and (H) quantitative analysis (n = 4). Western blot analysis of (I) Arg and iNOS expression in tumor tissues after various treatments, and the quantitative analysis of (J) Arg and (K) iNOS protein levels (n = 4). (L) CD3^+^CD8^+^ immunofluorescence images of tumor tissues isolated from Hepa1‐6 tumor‐bearing mice after various treatments. Scale bar: 100 µm. Statistical analysis between the indicated groups was performed using one‐way ANOVA analysis. *p < 0.05, **p < 0.01, ***p < 0.001, ****p < 0.0001.

Subsequently, an antitumor study was conducted to determine whether αPDL1‐VLPs/shPCSK9 could effectively suppress tumor growth. The treatment regimen was illustrated in Figure S17. Mice were randomly assigned to three groups receiving saline, αPDL1‐VLPs, or αPDL1‐VLPs/shPCSK9 treatment. On the 13th day, the αPDL1‐VLPs group exhibited a 56.1% inhibition rate, with a tumor volume of 308.3 ± 137.4 mm^3^, compared to 702.0 ± 204.3 mm^3^ in the untreated group (Figure 4B,C). This suppression was likely attributed to the immune checkpoint blockade effect of the PD‐L1 nanobody. Additionally, αPDL1‐VLPs/shPCSK9 not only served as a drug carrier to facilitate shPCSK9 delivery but also acted as an immunostimulatory agent to enhance antitumor efficacy. Moreover, the tumor volume growth rate in the αPDL1‐VLPs/shPCSK9 treatment group was significantly and uniformly slowed down (Figure S18). The αPDL1‐VLPs/shPCSK9 group showed a marked reduction in tumor volume (213.0 ± 71.5 mm^3^), achieving an inhibition rate of 69.6%. Besides, tumor excision and subsequent analysis revealed significantly smaller tumor sizes in the αPDL1‐VLPs/shPCSK9 group compared to the other groups (Figure 4D). The average tumor weight in the untreated group was 0.7 g, approximately 3.7 times higher than that in the αPDL1‐VLPs/shPCSK9 group, demonstrating its superior therapeutic efficacy (Figure 4E). Histological analysis further confirmed these findings. H&E staining revealed extensive apoptotic and necrotic tumor cells in the αPDL1‐VLPs/shPCSK9 group. Futhermore, αPDL1‐VLPs/shPCSK9 effectively induced tumor cell death and inhibited proliferation, as demonstrated by the strongest apoptotic signal observed in TUNEL staining and minimal proliferative activity indicated by Ki67 staining (Figure 4F). Importantly, no distinct changes in body weight were observed throughout the treatment, indicating the low systemic toxicity of αPDL1‐VLPs/shPCSK9 (Figure S19).

Next, to confirm the regulatory effect of αPDL1‐VLPs/shPCSK9 on PCSK9 protein expression in vivo, tumor tissues were lysed and analyzed via western blotting. As shown in Figure 4G, αPDL1‐VLPs/shPCSK9 dramatically inhibited PCSK9 expression in tumors, with statistically significant differences observed compared to both the non‐treatment and αPDL1‐VLPs groups (Figure 4H). By suppressing PCSK9, αPDL1‐VLPs/shPCSK9 effectively disrupted the cholesterol metabolism of tumor cells, reducing the cholesterol required for cell proliferation and subsequently inhibiting tumor growth. Additionally, the phenotypic transformation of macrophages induced by αPDL1‐VLPs/shPCSK9 was assessed in vivo (Figure 4I). As shown in Figure 4J,K, αPDL1‐VLPs/shPCSK9 downregulated Arg and upregulated iNOS, confirming the conversion of macrophages to the antitumor M1 phenotype. Moreover, the αPDL1‐VLPs/shPCSK9‐treated group exhibited abundant T cell infiltration in the tumor (Figure 4L and S20). In a word, αPDL1‐VLPs/shPCSK9 not only directly suppressed tumor growth by interfering with PCSK9 but also exerted immunomodulatory effects by polarizing macrophages and promoting T cell infiltration.

### 2.7 Immune Activation Characteristics of αPDL1‐VLPs/shPCSK9 In Vivo

To investigate the potential of αPDL1‐VLPs/shPCSK9 to eradicate distant tumors, the immune cell populations in the spleen and tumor tissues were analyzed. The gating strategies and corresponding cell numbers were shown in Figures S22‐S24. As shown in Figure 5A,B, mice treated with αPDL1‐VLPs alone did not exhibit significant changes in the percentage of CD3^+^CD4^+^ helper T cells (Th cells) and CD3^+^CD8^+^ CTLs in the spleen. However, treatment with αPDL1‐VLPs/shPCSK9 distinctly increased the percentage of CTLs and the CD8^+^/CD4^+^ T cells ratio in the spleen, indicating that downregulation of PCSK9 promoted the activation of antitumor immunity (Figure 5C). Compared to the control group (Blank), the proportion of Th cells at the tumor site was significantly reduced in the αPDL1‐VLPs/shPCSK9 treatment group, from 51.9% in the control group to 28.5% (Figure 5D). In contrast, the proportion of CTLs increased markedly to 52.1% (Figure 5E). These results clearly demonstrated that treatment with αPDL1‐VLPs/shPCSK9 substantially remodeled the tumor immune microenvironment, leading to CD8^+^ cytotoxic T cells becoming the dominant cell population (Figure 5F). This shift directly supported the proposed mechanism underlying the tumor‐clearing effect of αPDL1‐VLPs/shPCSK9 and aligned well with previous observations of tumor growth inhibition. Additionally, the percentage of tumor‐infiltrating dendritic cells (DCs) increased in both the αPDL1‐VLPs and αPDL1‐VLPs/shPCSK9 groups, suggesting enhanced activation of antigen‐presenting cells (Figure 5G). Importantly, the treatment also induced a shift in macrophages from the M2 immunosuppressive phenotype to the M1 antitumor phenotype (Figure 5H,I). In the untreated group, the M2‐to‐M1 macrophage ratio was 3.7, whereas in the αPDL1‐VLPs/shPCSK9 treatment group, the ratio significantly decreased to 1.4 (Figure 5J). This phenotypic reversal not only directly weakened the immunosuppressive barrier within the tumor microenvironment but also synergistically enhanced the effector function of CD8^+^ cytotoxic T cells, thereby collectively amplifying the antitumor immune response (Figure 5K).

**FIGURE 5:**
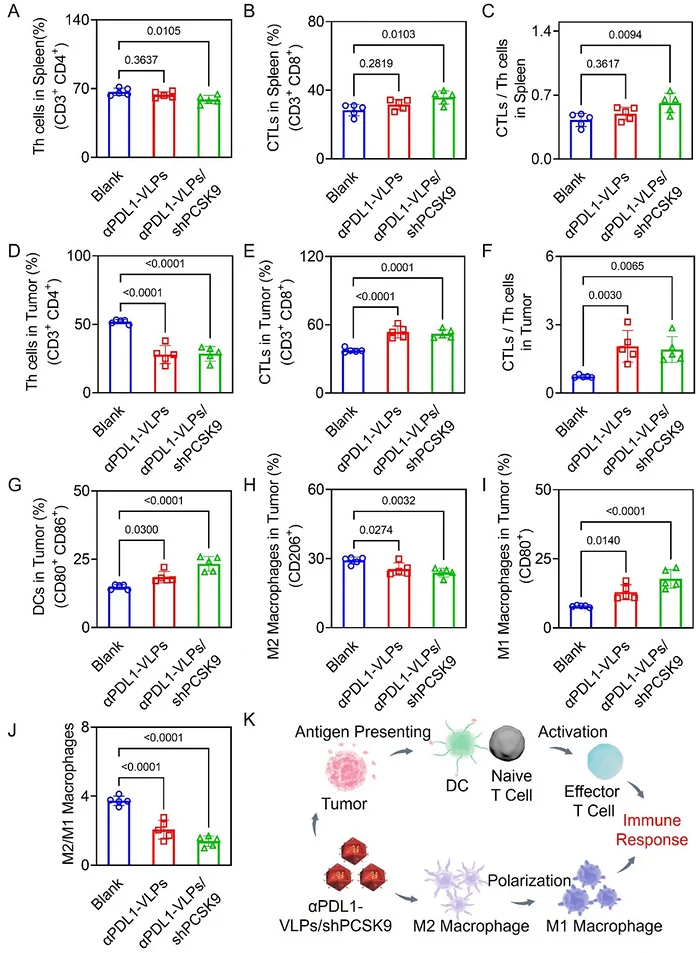
Immune activation effects of αPDL1‐VLPs/shPCSK9. Quantitative analysis of (A) Th cells marked with CD3^+^CD4^+^, (B) CTLs marked with CD3^+^CD8^+^, and (C) the ratio of CTLs to Th cells in spleens after treatment with αPDL1‐VLPs, αPDL1‐VLPs/shPCSK9 (n = 5). Quantitative analysis of (D) Th cells marked with CD3^+^CD4^+^, (E) CTLs marked with CD3^+^CD8^+^, (F) the ratio of CTLs to Th cells, (G) dendritic cells (DCs) marked with CD80^+^CD86^+^, (H) M2 macrophages marked with CD206^+^, (I) M1 macrophages marked with CD80^+^ and (J) the ratio of M2 to M1 macrophages in tumors after treatment with αPDL1‐VLPs, αPDL1‐VLPs/shPCSK9 (n = 5). (K) Schematic diagram of the immune response mediated by αPDL1‐VLPs/shPCSK9 Statistical analysis between the indicated groups was performed using one‐way ANOVA analysis. *p < 0.05, **p < 0.01, ***p < 0.001, ****p < 0.0001.

### 2.8 Distant Tumor Inhibition Effects of αPDL1‐VLPs/shPCSK9

Benefiting from its systemic immune activation, the abscopal effects of αPDL1‐VLPs/shPCSK9 were evaluated in a distant tumor model. In this model, the right tumor was intratumorally injected with various agents, while the left tumor remained untreated (Figure S21). As shown in Figure 6A, no obvious therapeutic differences were observed in the growth of the intratumorally injected primary tumors until day 9, when untreated tumors began to exhibit rapid growth. On day 13, tumors in the αPDL1‐VLPs and αPDL1‐VLPs/shPCSK9 groups showed suppressed growth, of which the latter group demonstrated a more pronounced effect with a tumor inhibition rate of 62.7% (Figure 6B). After harvesting tumors from mice, αPDL1‐ VLPs/shPCSK9 inhibited tumor growth, showing a mean tumor weight of 0.139 g, which was 0.36 times that of untreated controls (Figure 6C,G). Notably, intratumoral injection not only effectively suppressed primary tumor growth but also resulted in remarkable inhibition of the untreated distant tumors. Longitudinal monitoring revealed dramatic therapeutic divergence by day 13, with αPDL1‐VLPs/shPCSK9 limiting tumors to 59.8 ± 7.4 mm^3^, whereas controls progressed to 170.6 ± 25.4 mm^3^ (Figure 6D). Furthermore, αPDL1‐VLPs and αPDL1‐VLPs/shPCSK9 treatments both significantly suppressed growth, exhibiting inhibition rates of 49.1% and 64.9%, respectively, relative to the non‐treatment group (Figure 6E). Therapeutic assessment of distant tumors revealed that αPDL1‐VLPs/shPCSK9 treatment induced a 0.31‐fold reduction in tumors compared to the control group (Figure 6F,H). This phenomenon could be attributed to the ability of αPDL1‐VLPs/shPCSK9 to induce systemic immune activation, resulting in the immune‐mediated elimination of distant tumors. Importantly, the direct intratumoral injection did not result in weight loss in the mice, indicating the good biocompatibility of αPDL1‐VLPs/shPCSK9 (Figure 6I). Subsequently, an immunological analysis of distant tumor tissue was performed to elucidate the underlying mechanism. As anticipated, treatment with αPDL1‐VLPs/shPCSK9 could induce profound systemic immune activation. Compared to the untreated control group, αPDL1‐VLPs/shPCSK9 treatment significantly increased tumor‐infiltrating Th cells by 1.4‐fold, boosted CTLs 1.3‐fold, and expanded DCs population 1.6‐fold (Figure 6J–L). Concurrently, M2 macrophages infiltration decreased by 18.9%, while the M1 macrophages proportion remained statistically unchanged. This coordinated immune reprogramming resulted in an 18.7% reduction of the protumorigenic M2/M1 macrophages ratio (Figure 6M–O). These findings strongly suggested that αPDL1‐VLPs/shPCSK9 treatment not only suppressed primary tumors but also inhibited untreated distant tumors through the activation of systemic antitumor immune responses.

**FIGURE 6:**
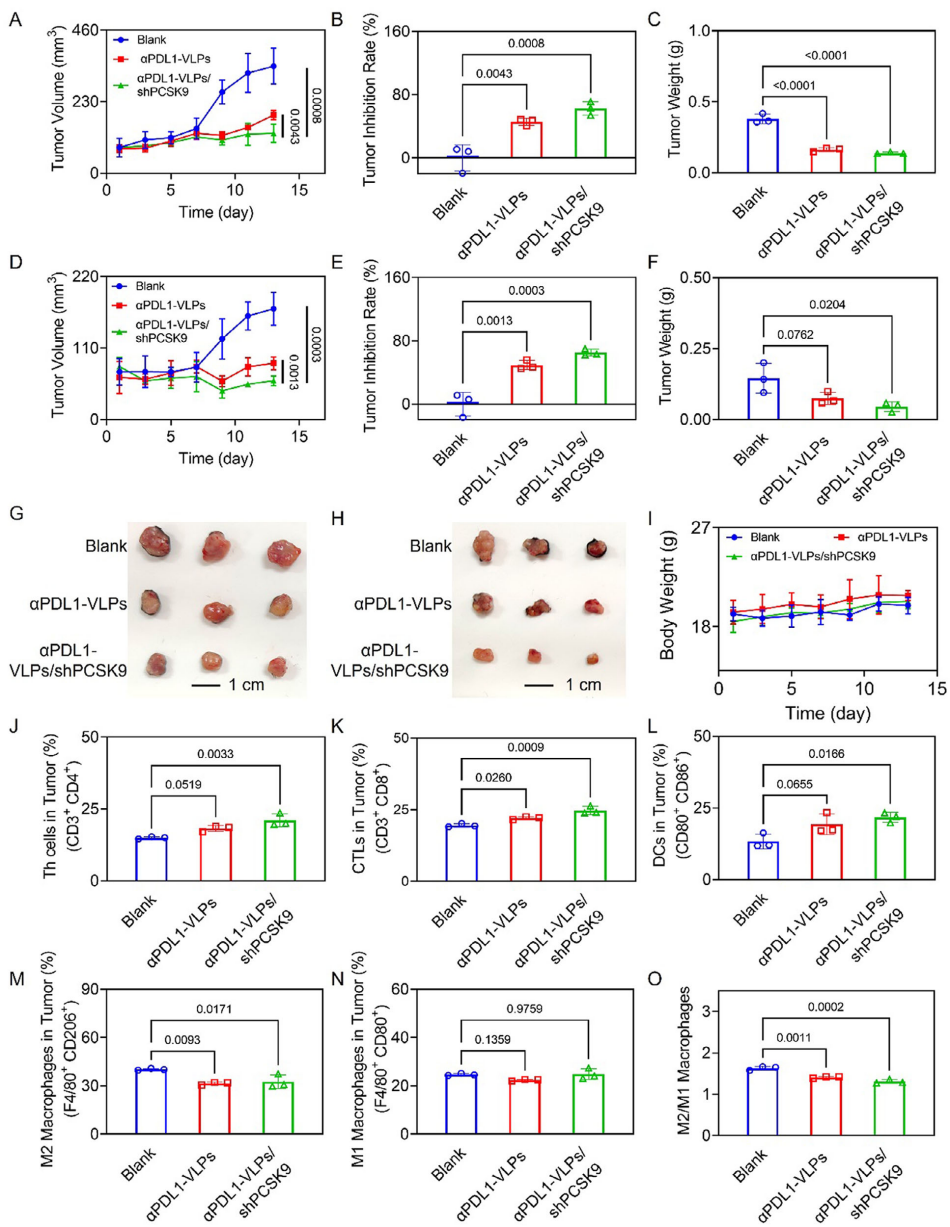
Abscopal effect of αPDL1‐VLPs/shPCSK9. (A) Primary tumor volume curves, (B) inhibition rate on day 13, and (C) ex vivo tumor weight after treatment with αPDL1‐VLPs or αPDL1‐VLPs/shPCSK9 (n = 3). (D) Distant tumor growth curve, (E) inhibition rate on day 13, and (F) *ex vivo* tumor weight of distant tumor after various treatments (n = 3). The sacrificed tumor tissues of (G) primary and (H) distant tumors with different treatments (n = 3). (I) Changes in body weight of mice following treatment with αPDL1‐VLPs or αPDL1‐VLPs/shPCSK9. Quantitative analysis of (J) Th cells labeled by CD3^+^CD4^+^, (K) CTLs labeled by CD3^+^CD8^+^, (L) DCs labeled by CD80^+^CD86^+^, (M) M2 macrophages labeled by F4/80^+^CD206^+^, (N) M1 macrophages labeled by F4/80^+^CD80^+^, and (O) the M2 to M1 macrophages ratio in distant tumors post‐treatment with αPDL1‐VLPs or αPDL1‐VLPs/shPCSK9 (n = 3). Statistical analysis between the indicated groups was performed using one‐way ANOVA analysis. *p < 0.05, **p < 0.01, ***p < 0.001, ****p < 0.0001.

### 2.9 Biosafety of αPDL1‐VLPs/shPCSK9

Despite the superior antitumor efficiency of αPDL1‐VLPs/shPCSK9 in vivo, its biosafety was also an important evaluation criterion. To test the biosafety of αPDL1‐VLPs/shPCSK9, major organs (heart, liver, spleen, lungs, and kidneys) were analyzed by H&E staining. No evident pathological damage was observed in either the αPDL1‐VLPs or αPDL1‐VLPs/shPCSK9 treatment group, suggesting minimal organ toxicity for these therapeutic agents (Figure 7A). Additionally, the results of blood biochemical markers indicated no significant differences in levels of alanine aminotransferase (ALT), aspartate aminotransferase (AST), blood urea nitrogen (BUN), and uric acid (UA) among the treatment groups, further confirming the safety of these agents concerning liver and kidney function (Figure 7B). Hemolysis assay results showed that even at concentrations up to 600 µg mL^−1^, αPDL1‐VLPs/shPCSK9 did not cause distinct red blood cell lysis, with hemolysis rates close to that of the negative control (PBS) compared to the positive control (deionized water) (Figure 7C). Even more crucially, endotoxin contamination testing indicated no obvious endotoxin contamination in the VLPs used, which avoided excessive immune responses that led to inflammation (Figure 7D). Overall, αPDL1‐VLPs and αPDL1‐VLPs/shPCSK9 possessed good biosafety in vivo, making them suitable potential candidates for HCC therapy.

**FIGURE 7:**
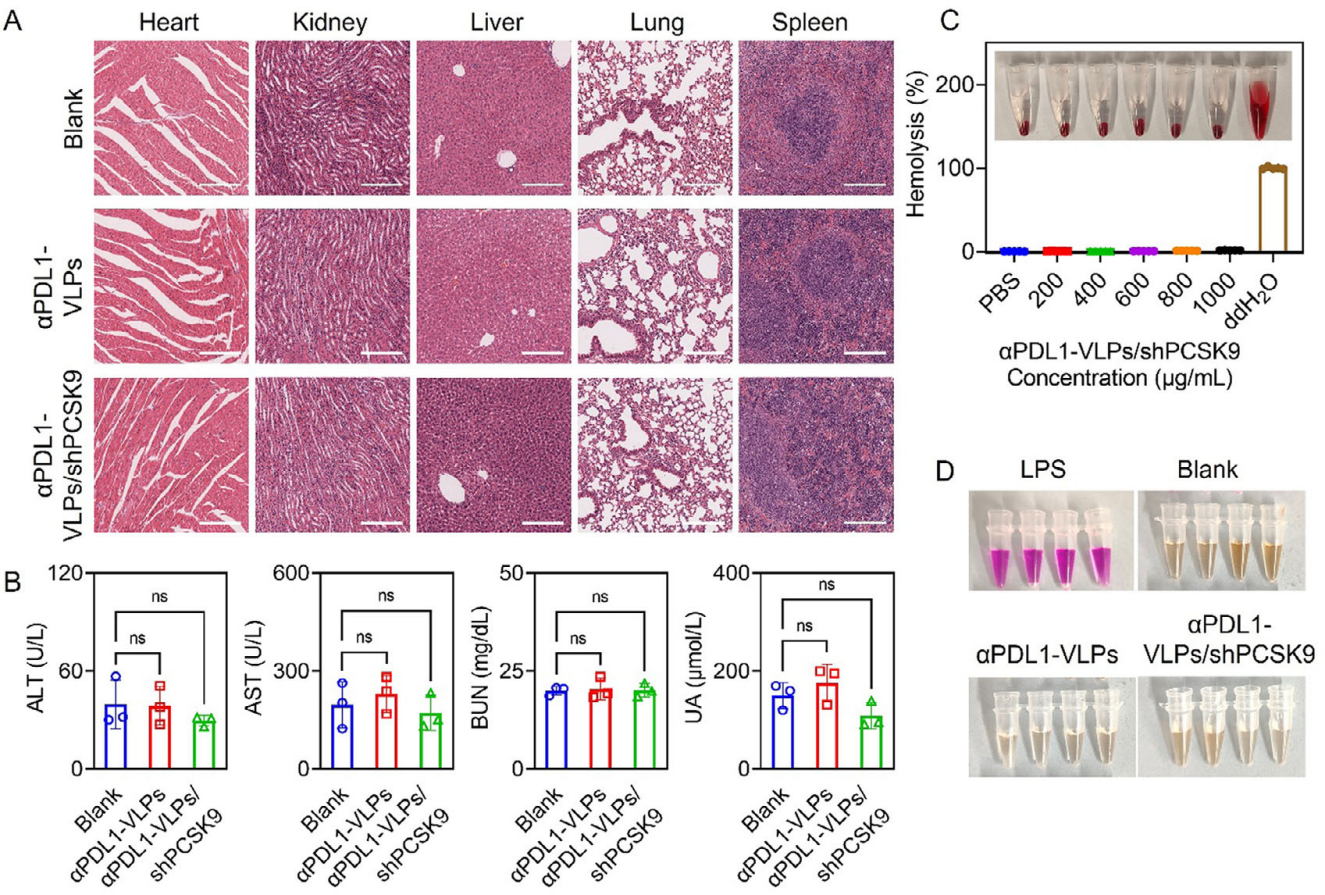
Biosafety analysis of αPDL1‐VLPs/shPCSK9. (A) H&E staining of heart, kidney, liver, lung and spleen following treatment with αPDL1‐VLPs, αPDL1‐VLPs/shPCSK9. Scale bar: 200 µm. (B) Blood biochemical analysis of ALT, AST, BUN, UA of the mice with different treatments on the 13th day (n = 3). (C) The hemolytic effects of different concentrations of αPDL1‐VLPs/shPCSK9, with PBS as the negative control and H_2_O as the positive control (n = 5). (D) Endotoxin analysis of treatments with αPDL1‐VLPs, or αPDL1‐VLPs/shPCSK9, with LPS as the positive control (n = 4). Statistical analysis between the indicated groups was performed using one‐way ANOVA analysis. ns meant non‐significant.

## 3 Conclusion

In summary, leveraging bioinformatic evidence associating elevated PCSK9 with impaired T cell function and poor HCC prognosis, we developed a one‐step synthetic RNA virus of αPDL1‐VLPs/shPCSK9 for HCC immunotherapy. Mechanistically, αPDL1‐VLPs/shPCSK9 could interfere with PCSK9 and upregulate MHC‐I, improving tumor cell recognition and increasing susceptibility to CTLs. Additionally, αPDL1‐VLPs/shPCSK9 promoted the polarization of macrophages from the immunosuppressive M2 phenotype to the pro‐inflammatory M1 phenotype, further boosting immune activation. In vivo studies demonstrated that αPDL1‐VLPs/shPCSK9 effectively accumulated in HCC tumors and suppressed primary tumor growth without causing systemic side effects. Immunological analysis confirmed that αPDL1‐VLPs/shPCSK9 enhanced CTLs activation and infiltration, initiating systemic antitumor immunity that eradicated distant tumors. Collectively, these findings highlighted αPDL1‐VLPs/shPCSK9 as a promising therapeutic strategy for overcoming the immunosuppressive microenvironment, offering a streamlined yet comprehensive approach to enhancing HCC immunotherapy.

## 4 Experimental Section

Experimental details are provided in the Supporting Information.

## Author Contributions

**Chuyu Huang**: methodology, investigation, writing – original draft. **Yun Ye**: methodology, data curation. **Yi Guo**: formal analysis. **Guozheng Liao**: investigation. **Linyi Luo**: supervision. **Lingwen Ding**: methodology. **Yingling Miao**: writing – review and editing. **Linping Zhao**: funding acquisition, writing – review and editing. **Yongchang Ouyang**: Conceptualization, Supervision. **Shiying Li**: Conceptualization, Writing – review and editing, Funding acquisition.

## Ethics Statement

All animal experiments were conducted in accordance with the Institutional Animal Care and Use Committee (IACUC) guidelines at the Guangzhou Medical University Animal Experiment Center. The research was authorized with project number GY2024‐182.

## Conflicts of Interest

The authors declare no conflicts of interest.

## Supporting information




**Supporting File 1**: exp270226‐sup‐0001‐SuppMat.docx.

## Data Availability

The data that support the findings of this study are available from the corresponding author upon reasonable request.
