# Supplementary material for: A PD‐L1 Nanobody‐Engineered Synthetic RNA Virus Enhances Hepatocellular Carcinoma Immunotherapy by Interfering PCSK9 and Upregulating MHC‐I
Source: Exploration (Beijing). 2026 Oct 6;6(5):70226. doi: 10.1002/exp2.70226 (PMC13643016; doi:10.1002/exp2.70226)
Supplement: Supplementary file 1 [file EXP2-6-70226-s001.docx]

Supporting Information

**A PD-L1 Nanobody-Engineered Synthetic RNA Virus Enhances Hepatocellular Carcinoma Immunotherapy by Interfering PCSK9 and Upregulating MHC-I**

**Experimental Section**

**Bioinformatics Analysis**

PCSK9 expression levels were obtained from the TIMER2.0 database (http://timer.cistrome.org/). Statistical significance was assessed using the Wilcoxon test, with significance indicated by asterisks (**P* < 0.05, ***P*< 0.01, ****P* < 0.001). For immune cell infiltration data, the TCGA-LIHC expression matrix was downloaded from the UCSC Xena database (https://xena.ucsc.edu/). The proportion of immune cells in each sample was calculated using the GSVA algorithm (https://www.bioconductor.org/packages/release/bioc/vignettes/GSVA/inst/doc/GSVA.html). The results were assessed using the Wilcoxon test (**P* < 0.05, ***P* < 0.01, ****P* < 0.001, *****P* < 0.0001). Survival analysis data were sourced from the CAMOIP database (https://www.camoip.net). Differences between groups were statistically evaluated using the log-rank test.

**Plasmid Construction**

Four plasmids, αPD-L1, αPDL1-VLPs, #1 αPDL1-VLPs/shPCSK9, and #2 αPDL1-VLPs/shPCSK9 were synthesized by Beijing Tsingke Biotech Co., Ltd.

**Plasmid Transformation**

Plasmids were transformed into competent *E. coli* and plated on LB agar medium. Selected colonies were cultured in LB liquid medium, shaken at 220 rpm (37°C) until the OD600 reached 0.6-0.8, and then expanded into 500 mL of fresh medium. Protein expression was induced with IPTG or arabinose.

**Sample Purification**

After protein expression induction, bacterial cells were harvested by centrifugation, lysed by ultrasonic disruption, and purified by AKTA Avant chromatography system. The purified product was dialyzed continuously at 4°C for 12 hours against PBS (pH 7.4), with the buffer replaced every 6 hours to remove residual impurities. Following dialysis, the product was stored at 4°C in the dark.

**Assessment of Sample Purity**

SDS-PAGE was performed to assess sample purity, followed by staining for protein visualization. Western blotting with a His-HRP antibody confirmed protein expression and purification.

**Nuclease Resistance Assay**

To assess nuclease resistance, purified samples and those treated with DNase I or RNase A were analyzed by gel electrophoresis.

**Morphological Observation**

The morphological characteristics of αPDL1-VLPs, #1 αPDL1-VLPs/shPCSK9, and #2 αPDL1-VLPs/shPCSK9 were captured using transmission electron microscopy at the Institute of Microbiology, Guangdong Academy of Sciences.

**ELISA Assay for Nanobody Binding to PD-L1 Protein**

PD-L1 protein was coated onto ELISA plates, followed by blocking with 5% BSA in PBS. Gradient dilutions of αPD-L1, αPDL1-VLPs, #1 αPDL1-VLPs/shPCSK9, and #2 αPDL1-VLPs/shPCSK9 samples were added and incubated. After washing, His antibody was added, incubated, and followed by addition of TMB substrate. The reaction was stopped, and the absorbance was measured at 450 nm.

**Cell Culture**

CT26 cells (mouse colorectal carcinoma cells), Hepa1-6 cells (mouse hepatocarcinoma cells), and 4T1 cells (mouse breast cancer cells) were cultured in high-glucose DMEM medium supplemented with 10% fetal bovine serum (FBS) and 1% penicillin-streptomycin. RAW264.7 cells (mouse mononuclear macrophage cells) were cultured in RPMI 1640 medium with the same concentrations of FBS and penicillin-streptomycin. All cells were maintained at 37°C in a humid atmosphere with 5% CO_2_.

**PD-L1 Expression in Different Cell Lines**

Total protein was extracted from CT26, Hepa1-6, and 4T1 cells, and PD-L1 expression was analyzed *via* Western Blot.

**Uptake of αPDL1-VLPs in Different Cell Lines**

CT26, Hepa1-6, and 4T1 cells were seeded into confocal dishes and incubated with cy5.5-labeled αPDL1-VLPs (αPDL1-VLPs@cy5.5) for 6 hours. After washing, cells were incubated with 10 μg mL^-1^ Hoechst 33342 for 15 minutes, then washed again. Uptake of αPDL1-VLPs@cy5.5 was observed using confocal laser scanning microscopy (CLSM) and quantified by flow cytometry.

**Uptake of αPDL1-VLPs After Targeted Blocking**

For targeted blocking, the experimental group was treated with PD-L1 monoclonal antibody (80 μg mL^-1^) for 12 hours, while the control group received no antibody. Both groups were then incubated with αPDL1-VLPs@cy5.5 for 4 hours. After washing and Hoechst staining, uptake was observed by CLSM and quantified by flow cytometry.

**PCSK9 Expression Analysis**

Hepa1-6 cells were treated with 400 μg mL^-1^ of αPDL1-VLPs, #1 αPDL1-VLPs/shPCSK9 and #2 αPDL1-VLPs/shPCSK9, respectively, for 24 hours. After incubation, total RNA was isolated from cultured cells using TRIzol reagent and subsequently reverse-transcribed into cDNA for RT-PCR analysis. In parallel, total protein was extracted from the cells, and PCSK9 protein expression was analyzed using western blot.

**Cytotoxicity of αPDL1-VLPs/shPCSK9**

Hepa1-6 cells were treated with serial dilutions of αPDL1-VLPs, #1 αPDL1-VLPs/shPCSK9, and #2 αPDL1-VLPs/shPCSK9 (up to 267 μg mL^-1^) for 48 hours. After incubation, 10% CCK8 solution was added, and cell viability was assessed by measuring optical density at 450 nm using a microplate reader.

**Uptake of αPDL1-VLPs/shPCSK9 in Hepa1-6 Cells**

Hepa1-6 cells were incubated with different concentrations of cy5.5-labeled #1 αPDL1-VLPs/shPCSK9 (αPDL1-VLPs/shPCSK9@cy5.5) for 3 or 9 hours. After incubation, the cells were washed and stained with Hoechst 33342. Then, the uptake of the labeled particles was observed using CLSM, and quantitative analysis was performed using flow cytometry.

**MHC-I Detection**

Hepa1-6 cells were treated with 400 μg mL^-1^ of αPDL1-VLPs or #1 αPDL1-VLPs/shPCSK9 for 24 hours. For CLSM, cells were fixed with 4% paraformaldehyde, permeabilized with 0.1% Triton X-100, and blocked with 5% goat serum. They were incubated overnight with Anti-MHC-I antibody, followed by incubation with Goat Anti-Rabbit IgG H&L and DAPI staining. Imaging was performed after a final 15-minute incubation. For flow cytometry, cells were washed, digested with trypsin, and stained with MHC-I-FITC antibody. MHC-I expression was analyzed by flow cytometry.

**Macrophage Polarization**

RAW264.7 cells were treated with 100 ng mL^-1^ IL-4 for 48 hours to induce their differentiation into M2 macrophages. The M2 macrophages were then treated with 200 μg mL^-1^ of αPDL1-VLPs and #1 αPDL1-VLPs/shPCSK9, respectively, for 24 hours. After treatment, the polarization status of the cells was assessed using western blot and flow cytometry.

**Animal Experiments**

All animal experiments were conducted in accordance with the Institutional Animal Care and Use Committee (IACUC) guidelines at the Guangzhou Medical University Animal Experiment Center. The research was authorized with project number GY2024-182 and involved female C57 mice aged 6 to 8 weeks.

**Fluorescence Imaging**

To assess drug accumulation *in vivo*, cy5.5 was used for fluorescence analysis. Hepa1-6 cells were implanted in the right legs of mice, and fluorescence imaging was performed once tumors reached the desired size. αPDL1-VLP/shPCSK9@cy5.5 was administered *via* tail vein injection, and fluorescence was monitored at specific time points. At the end of the experiment, mice were dissected, and fluorescence imaging of major organs and tumors was performed.

**Tumor Inhibition Experiments**

To evaluate the antitumor efficacy of #1 αPDL1-VLPs/shPCSK9, a Hepa1-6 tumor-bearing mouse model was established by injecting Hepa1-6 cells into the right hind leg of mice. Seven days later, mice received tail vein injections of αPDL1-VLPs (25 mg kg^-1^) or #1 αPDL1-VLPs/shPCSK9 (25 mg kg^-1^) every two days for five treatments. Tumor volume and body weight were monitored every two days throughout the 13-day experiment. On Day 13, mice were euthanized, and blood serum was collected for biochemical analysis. Major organs were analyzed using H&E staining, and tumor tissues were examined with H&E, TUNEL, Ki67, and CD3^+^CD8^+^ T cell staining.

**Hemolysis Assay**

Mouse venous blood was collected in EDTA-containing tubes and mixed gently to prevent clotting. The blood was centrifuged at 1500 rpm for 10 minutes to separate plasma and white blood cells, leaving red blood cells. The red blood cells were washed three times with saline, centrifuging each time to obtain a pellet. PBS was used as a negative control, ultrapure water as a positive control, and various concentrations of #1 αPDL1-VLPs/shPCSK9 as the experimental group. The red blood cell pellet was resuspended in each treatment group to a final concentration of 2% and incubated at 37°C for 1 hour. After centrifugation, the supernatant absorbance at 540 nm was measured to calculate the hemolysis rate.

**Endotoxin** **Analysis**

After drug treatment in tumor-bearing mice, venous blood was collected using endotoxin-free equipment. Blood from untreated mice served as the negative control, and LPS was used as the positive control. Endotoxin detection reagents were added to the samples, which were incubated at 37°C for 30 minutes. A chromogenic solution was then added, and the reaction continued for 5 minutes at 37°C in the dark. After adding three buffers and mixing, absorbance was measured at 545 nm using a microplate reader.

**Analysis of Immune Cells**

On Day -7, Hepa1-6 cells were injected into the right hind leg of mice to establish a tumor model. On Days 1, 3, 5, 7, and 9, αPDL1-VLPs (25 mg kg^-1^) and #1 αPDL1-VLPs/shPCSK9 (25 mg kg^-1^) were administered via tail vein injection. On Day 10, the mice were sacrificed, and the spleens and tumor tissues were collected for immune cell analysis. Spleens were ground, flushed with HBSS, and filtered through a 40 μm mesh. Subsequently, the samples were lysed with red blood cell lysis buffer for 10 minutes, followed by washing with PBS. The cell suspension was centrifuged at 1200 rpm for 5 minutes at 4°C and stained with flow cytometry antibodies to analyze the proportions of Th cells (CD4^+^ T cells) and CTLs (CD8^+^ T cells). For the preparation of single-cell suspensions from tumor tissues, freshly isolated tumor tissues were minced into a homogenate. The minced tissues were then digested in a 37°C with a solution containing 0.1% collagenase IV, 0.01% hyaluronidase, and 0.05% DNase I. Digestion was performed twice for 30 minutes each time. All supernatants were combined and filtered through a 40 μm sterile cell sieve to remove undigested tissue debris. For T cell subset staining (Th cells, CTLs), the steps were as follows. First, viability dyes were used for staining at 37°C for 10 minutes. Subsequently, antibodies CD45-V500, CD3-APC, CD4-RB705, and CD8-FITC were added at the recommended doses, followed by incubation on ice for 45 minutes. For DC subset staining, the steps were as follows. First, viability dyes were used for staining at 37°C for 10 minutes. Next, antibodies CD45-V500, CD11c-ef450, CD80-FITC, and CD86-PE were added at the recommended dose, followed by incubation on ice for 45 minutes. For macrophage subset staining (M1, M2), the staining protocol for viability dyes, CD45-V500, CD11b-RB705, and CD80-FITC was the same as described above. Then, the cells were fixed and permeabilized, followed by the addition of intracellular antibody CD206-PE, and incubation on ice for 45 minutes. The gating strategies for all immune cell subsets were fully presented in Figure S22 and Figure S23, and the total numbers of DCs and CD8^+^CD3^+^ T cells in TILs were shown in Figure S24.

**Suppression of Distant Tumors**

On day -4, Hepa1-6 cells were inoculated into the right leg (primary tumor), and on day -2, into the left leg (distant tumor). On day 1, αPDL1-VLPs (18.7 mg kg^-1^) and #1 αPDL1-VLPs/shPCSK9 (18.7 mg kg^-1^) were intratumorally injected into the primary tumor. Treatment was repeated every two days for five doses. Body weight and tumor volume were measured every two days for 13 days. On day 13, both primary and distant tumors were collected for photography, weighing, and immune cell analysis.

**Statistical Analysis**

All results were presented as mean ± s.d. One-way analysis of variance (ANOVA) was used to analyze differences among multiple groups. Two-tailed Student’s t-test was used to analyze differences between two groups. All analyses were conducted using GraphPad Prism 9.5. The threshold used for statistical significance was *P* < 0.05.

**Figure S1.** The plasmid construction of (A) αPDL1 and (B) αPDL1-VLPs.

**Figure S2.** (A) Single colonies of *E. coli* harboring the plasmid carrying the αPDL1-VLPs genetic construct. (B) Metabolite collected from *E. coli* separated and purified by nickel affinity chromatography to isolate αPDL1-VLPs. a-d: Flow-through peak of metabolite without His-tags that could not bind to the nickel column; e: Elution peak containing His-tagged αPDL1-VLPs. (C) The presence of A protein (AP), coat protein-PDL1 nanobody-His tag (CP-Nb-His) and coat protein (CP) in αPDL1-VLPs though SDS-PAGE staining. 1: *E. coli* containing the αPDL1-VLPs plasmid but without the initiation of αPDL1-VLPs protein translation and assembly. 2: αPDL1-VLPs expressed and assembled in *E. coli*, followed by purification. M: PM5100 marker. (D) Western blot image with His-tag of αPDL1-VLPs before and after induced expression and purification in *E. coli*. 1: *E. coli* containing the αPDL1-VLPs plasmid but without the initiation of αPDL1-VLPs protein translation and assembly. 2: αPDL1-VLPs expressed and assembled in *E. coli*, followed by purification. M: PM5100 marker.

**Figure S3.** (A) Western blot image of αPDL1-VLPs with Bacteriophage MS2 antibody. Marker: PM5100. (B) The number of CP containing αPD-L1 in αPDL1-VLPs.

**Figure S4.** (A) Evaluation of immune cell composition in HCC patients stratified by low and high PCSK9 expression levels. (B) Kaplan-Meier survival curves showing overall survival differences between LIHC patients with low and high PCSK9 expression. Statistical analysis between the indicated groups was performed using Wilcoxon test (A) and log-rank test (B). **P* < 0.05, ***P* < 0.01, ****P* < 0.001, *****P* < 0.0001.

**Figure S5.** The plasmid construction of #1 αPDL1-VLPs/shPCSK9 and #2 αPDL1-VLPs/shPCSK9.

**Figure S6.** (A) Single colonies of *E. coli* harboring the plasmid carrying the #1 αPDL1-VLPs/shPCSK9 genetic construct. (B) Metabolite collected from *E. coli* separated and purified by nickel affinity chromatography to isolate #1 αPDL1-VLPs/shPCSK9. a-d: Flow-through peak of metabolite without His-tags that could not bind to the nickel column; e: Elution peak containing His-tagged #1 αPDL1-VLPs/shPCSK9. (C) Single colonies of *E. coli* harboring the plasmid carrying the #2 αPDL1-VLPs/shPCSK9 genetic construct. (D) Metabolite collected from *E. coli* separated and purified by nickel affinity chromatography to isolate #2 αPDL1-VLPs/shPCSK9. a-d: Flow-through peak of metabolite without His-tags that could not bind to the nickel column; e: Elution peak containing His-tagged #2 αPDL1-VLPs/shPCSK9.

**Figure S7.** The presence of AP, CP-Nb-His and CP in #1 αPDL1-VLPs/shPCSK9 (left) and #2 αPDL1-VLPs/shPCSK9 (right) though SDS-PAGE staining. 1: *E. coli* containing the #1 αPDL1-VLPs/shPCSK9 plasmid but without the initiation of #1 αPDL1-VLPs/shPCSK9 protein translation and assembly. 2: #1 αPDL1-VLPs/shPCSK9 expressed and assembled in *E. coli*, followed by purification. 3: *E. coli* containing the #2 αPDL1-VLPs/shPCSK9 plasmid but without the initiation of #2 αPDL1-VLPs/shPCSK9 protein translation and assembly. 4: #2 αPDL1-VLPs/shPCSK9 expressed and assembled in *E. coli*, followed by purification. M: PM5100 marker.

**Figure S8.** Hydrodynamic diameter of (A) #1 αPDL1-VLPs/shPCSK9 and (B) #2 αPDL1-VLPs/shPCSK9.

**Figure S9.** Western blot image with His-tag of #1 αPDL1-VLPs/shPCSK9 (left) and #2 αPDL1-VLPs/shPCSK9 (right). 1: *E. coli* containing the #1 αPDL1-VLPs/shPCSK9 plasmid but without the initiation of #1 αPDL1-VLPs/shPCSK9 protein translation and assembly. 2: #1 αPDL1-VLPs/shPCSK9 expressed and assembled in *E. coli*, followed by purification. 3: *E. coli* containing the #2 αPDL1-VLPs/shPCSK9 plasmid but without the initiation of #2 αPDL1-VLPs/shPCSK9 protein translation and assembly. 4: #2 αPDL1-VLPs/shPCSK9 expressed and assembled in *E. coli*, followed by purification. M: PM5100 marker.

**Figure S10.** Western blot images of (A) #1 αPDL1-VLPs/shPCSK9 and (B) #2 αPDL1-VLPs/shPCSK9 with bacteriophage MS2 antibody. Marker: PM5100.

**Figure S11.** Nuclease degradation resistance of (A) #1 αPDL1-VLPs/shPCSK9 and (B) #2 αPDL1-VLPs/shPCSK9. M: DL 5000 DNA marker; 1: #1 αPDL1-VLPs/shPCSK9; 2: #1 αPDL1-VLPs/shPCSK9 with DNase Ⅰ and RNase A; 3: #2 αPDL1-VLPs/shPCSK9; 4: #2 αPDL1-VLPs/shPCSK9 with DNase Ⅰ and RNase A.

**Figure S12.** Western blot analysis of PCSK9 protein expression in Hepa1-6 cells after treatment with #1 αPDL1-VLPs/shPCSK9, #2 αPDL1-VLPs/shPCSK9 and αPDL1-VLPs.

**Figure S13.** The hydrodynamic diameter of #1 αPDL1-VLPs/shPCSK9 at (A) day 7 and (B) day 14.

**Figure S14.** (A)Flow cytometry analysis and (B) quantitative analysis of cell apoptosis after treatment with Sorafenib, or αPDL1-VLPs/shPCSK9 (n=3). Statistical analysis between the indicated groups was performed using one-way ANOVA analysis. ****P* < 0.001, *****P* < 0.0001.

**Figure S15.** Biodistribution analysis of αPDL1-VLPs/shPCSK9@cy5.5 was conducted using real-time fluorescence imaging following intravenous administration to two distinct mice. 1-6 respectively represented the *ex vivo* images of heart, liver, spleen, lung, kidney and tumor tissues.

**Figure S16.** Quantitative fluorescence analysis of drugs in major organs and tumor after 24 hours (n=3).

**Figure S17.** Timeline schematic of tumor establishment and treatment.

**Figure S18.** Tumor volume curves of mice in each treatment group over a 13-day period (n=6).

**Figure S19.** Body weight variations of mice after treatment with αPDL1-VLPs, αPDL1-VLPs/shPCSK9 (n = 6).

**Figure S20.** The number of immunofluorescence-labeled CD3^+^CD8^+^ T cells in tumor tissues (n=4). Statistical analysis between the indicated groups was performed using one-way ANOVA analysis. ***P* < 0.01.

**Figure S21.** Schematic diagram of αPDL1-VLPs/shPCSK9 treatment on bilateral tumor model.

**Figure S22.** Gating strategy for flow cytometric analysis of immune cell populations in spleens.

**Figure S23.** Gating strategy for flow cytometric analysis of immune cell populations in tumor.

**Figure S24.** The total numbers of DCs and CD8^+^CD3^+^ T cells in single tumor model.
